# Research on Applications of Transurethral Plasmakinetic Enucleation of the Prostate in Elderly Patients Aged ≥80 Years

**DOI:** 10.3389/fsurg.2021.775548

**Published:** 2021-11-10

**Authors:** Wen Su, Ye Yi, Liang Zeng, Jin Tang

**Affiliations:** Department of Urology, Third Xiangya Hospital, Central South University, Changsha, China

**Keywords:** transurethral plasmakinetic enucleation, benign prostate hyperplasia, elderly people, transurethral resection of prostate, safe, efficacious

## Abstract

**Objective:** To evaluate the safety and efficacy of transurethral plasmakinetic enucleation of the prostate (PKERP) vs. transurethral resection of the prostate (TURP) in elderly patients aged ≥80 years with benign prostate hyperplasia.

**Materials and Methods:** We conducted a retrospective analysis of the PKERP (*n* = 123) and TURP (*n* = 143) in patients aged ≥80 years at urology department of The Third Xiangya Hospital of Central South University from January 2016 to October 2019. Then the preoperative, intraoperative, and postoperative data of different indicators were compared between the two groups. The follow-up was done at 3 months, 1 year after surgical treatment.

**Results:** No significant differences were observed between the two groups for the baseline characteristics, including age, prostate volume, prostate-specific antigen (PSA) level, concurrent disease, maximum urinary flow rate (MFR), international prostate symptoms score (IPSS), and quality of life (QoL) score. The operative time, hemoglobin decrease, and postoperative flushing time were significantly lower in the PKERP group compared with the TURP group. However, no significant differences were observed between both groups for postoperative hospital stay, incidence of transurethral resection syndrome (TURS), prostatic capsular perforation, and genuine urinary incontinence. The follow-up results showed that the MFR of the PKERP group was significantly higher than the TURP group at 1 year after surgery.

**Conclusion:** Compared with TURP, PKERP is a safe and efficacious method for treating patients aged ≥80 years with benign prostate hyperplasia, and it may improve long-term urination symptoms.

## Introduction

Benign prostate hyperplasia (BPH) is a common disease resulting in urination disorders in middle-aged and elderly men (1). It has a slow progression and aggravates with age (2). The prevalence of BPH reaches 83% in 80-year-olds (3). With improvement in the standards of living and health care and significantly increased average life expectancy in China, the number of elderly patients with BPH is increasing annually. It is particularly observed in elderly patients aged ≥80 years who may present with varying degrees of abnormalities or insufficiency in functions of the heart, lung, brain, and other major organs on consultation. This poses challenges for treatment selection by clinical staff. Therefore, treating urination disorders caused by BPH in elderly patients has become a challenge for urology staff in China. Transurethral resection of the prostate (TURP) is regarded as the golden standard to treat BPH for decades. Transurethral plasmakinetic enucleation of the prostate (PKERP) has recently emerged as a modified surgical method for treating BPH. There are accumulating evidence showing that while achieving satisfying surgical outcomes, this method can also decrease the risk of complications associated with TURP. However, for elderly patients, especially for patients aged ≥80 years with underlying health problems, the safety and efficacy of PKERP remain controversial. Therefore, we conducted this retrospective analysis to examine the safety and efficacy of PKERP in patients aged ≥80 years.

## Materials and Methods

We conducted a retrospective analysis of 266 male patients aged ≥80 years with a confirmed diagnosis of BPH at our department from January 2016 to October 2019 and surgical treatment. Surgical indications were in accordance with the 2019 edition of the China Urological Disease Diagnosis and Treatment Guidelines for BPH. In particular, these indications included patients with BPH and moderate or severe lower urinary tract symptoms (LUTS) that significantly affect the quality of life (QoL), poor treatment outcomes or intolerable adverse reactions with drugs, BPH resulting in ≥2 urine retention events, recurrent hematuria or urinary tract infection, comorbid bladder stones, and secondary hydronephrosis of the upper urinary tract. This study was approved by the IRB (Institutional Review Board) of the Third Xiangya Hospital of Central South University.

### Preoperative Data

Data collected included patient's age, underlying health problems, international prostate symptom score (IPSS), QoL scores, abridged 5-item version of the 15-item International Index of Erectile Function (IIEF-5) scores, transrectal ultrasound-guided prostate volume, and prostate-specific antigen levels. Considering that patients are of advanced age, preoperative urodynamic studies (UDS) were conducted for preliminary evaluation of surgery effects. Simultaneously, evaluation of heart and lung functions was conducted. If urinary tract infection was present preoperatively, urine culture plus drug sensitivity testing was conducted, followed by anti-infective treatment.

### Surgical Procedures

All patients signed the informed consent form and chose to undergo one-time spinal anesthesia.

**Figure 1:**
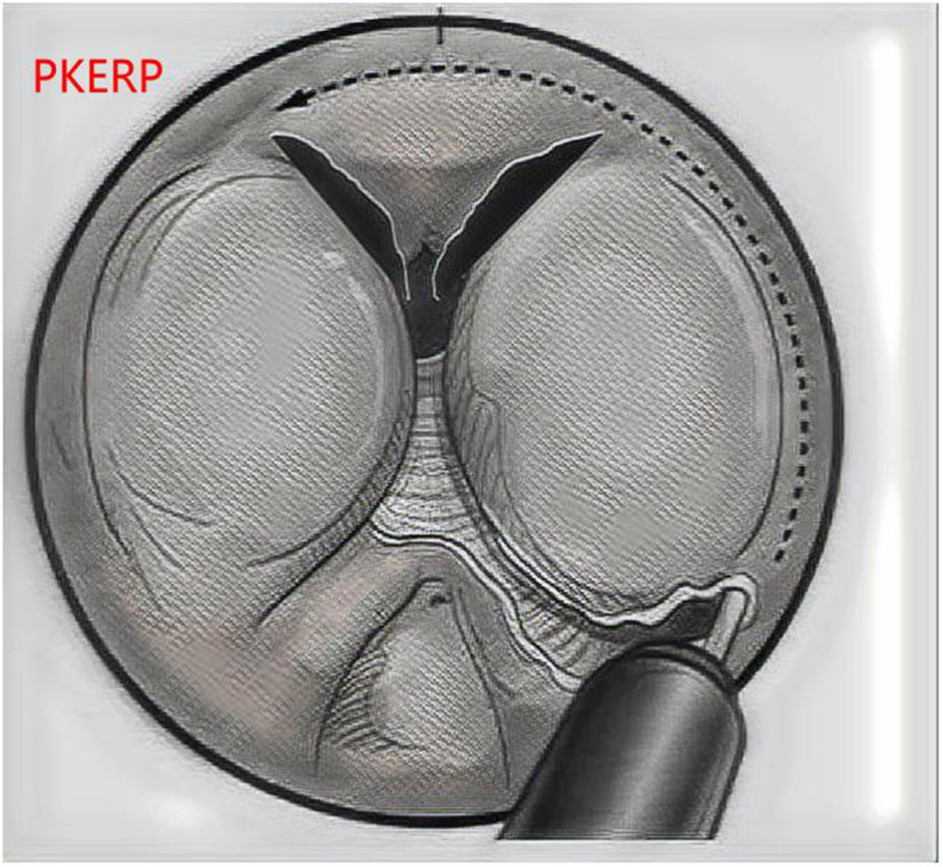
The PKERP surgical operation diagram.

Regarding the steps for PKERP (Figure 1), first, the sheath of the Fr-27 bipolar resectoscope was inserted to observe the prostatic urethra and bladder. Then, the left lobe of the prostate gland was stripped in a counterclockwise direction from the 5 o'clock position to the 1 o'clock position. Subsequently, the median lobe was stripped clockwise from the 5 o'clock position to the 7 o'clock position. The right lobe of the prostate was stripped clockwise from the 7 o'clock position to the 11 o'clock position. The top of the prostate gland (11–1 o'clock position) was sharply severed using a plasmakinetic cutting loop, and a 1-cm-long prostate tissue was retained to assist in urinary control. The entire lobe of the prostate tissue was pushed into the bladder along the capsule. Finally, a tissue morcellator (HAWK Inc, Hangzhou, China) was implanted to morcellate the prostate gland before suction.

**Figure 2:**
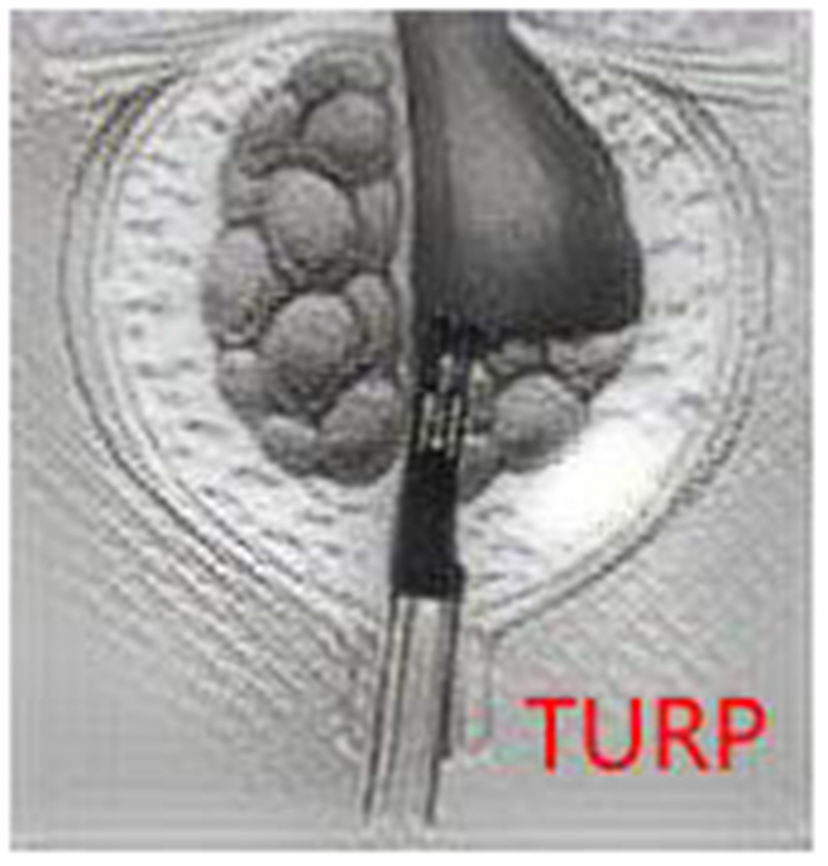
The TURP surgical operation diagram.

Transurethral resection of the prostate was performed from the bladder neck to the verumontanum, and then the anterior, lateral, and apical tissues were removed until the surgical capsule (Figure 2). Both the TURP and PKERP surgical procedures were performed by the same two skilled surgeons using the same equipment (Fr-27 bipolar resectoscope).

### Postoperative Management

Postoperatively, an Fr18 triple-lumen urinary catheter was inserted to continue bladder irrigation. On postoperative day 1, patients were encouraged to leave their beds. On day 2, levator ani muscle training was conducted. On postoperative day 3-5, the urinary catheter was removed, and the patient's urinary control and urination status was observed. Urine test and urine flow rate (UFR) test were conducted before discharge.

### Follow-Up Protocol

All patients underwent follow-up consultation at postoperative 3 and 12 months. Follow-up review includes IPSS, QoL, IIEF-5 scores, routine urine test, UFR, and postvoid residual volume measurements. During follow-up, patients with severe symptoms of urgency and frequency were administered solifenacin for 1 month. Solifenacin was gradually discontinued after these symptoms were controlled.

### Statistical Analysis

The Statistical Package for the Social Sciences (SPSS) 19.0 software was used for statistical analysis. Quantitative data were expressed using mean ± SD (*x* ±SD)), and qualitative data were expressed using *n* (%). The student's *t*-test performed statistical analysis or a chi-square test, and a difference of *P* < 0.05 was considered to be statistically significant.

## Results

### Preoperative Data

From January 2016 to October 2019, 266 elderly patients aged ≥80 years underwent prostate enucleation at our department, including PKERP (*n* = 123) and TURP (*n* = 143) (*p* > 0.05). The mean follow-up time was 12.9 *vs*. 13.6 months in PKERP group and TURP group, respectively (*p* > 0.05). Table 1 represents preoperative data. These 266 patients underwent preoperative urodynamic testing. in the PKERP and TURP groups were 59.2 and The mean maximum detrusor pressures 58.7 cmH_2_O (*p* > 0.05), respectively. Twenty-one patients had weak detrusor muscles (maximum detrusor muscle pressure <40 cm H_2_O during pressure and flow rate tests) in PKERP group and 22 in TURP group (*p* > 0.05). There were 25 patients in PKERP and 26 patients in TURP group who had indwelling urinary catheter placement preoperatively (*p* > 0.05). The mean prostate volume was 67.7 mL *vs*. 62.9 mL in PKERP and TURP group, respectively (*p* > 0.05).

**Table 1:** Preoperative data.

**Items**	**PKERP**	**TURP**	***P***
Number of patients	123	143	*p* > 0.05
Mean age (years)	83.8 ± 3.6	84.9 ± 5.4	*p* > 0.05
Mean follow-up (month)	12.9 ± 0.5	13.6 ± 0.7	*p* > 0.05
Qmax (mL/s)	7.4 ± 1.9	7.1 ± 1.5	*p* > 0.05
Pdet max maximum detrusor muscle pressure (cmH_2_O)	59.2 ± 37.9	58.7 ± 37.2	*p* > 0.05
tPSA	8.0 ± 7.8	8.7 ± 8.3	*p* > 0.05
Mean prostate volume (ml)	67.7 ± 24.4	62.9 ± 25.2	*p* > 0.05
Mean IPSS	20.4 ± 2.8	23.6 ± 2.3	*p* > 0.05
Mean QoL score	4.6 ± 0.4	4.7 ± 0.6	*p* > 0.05

### Intraoperative Status and Surgical Complications

No life-threatening complications occurred during surgery and postoperatively in all patients. In our group of patients undergoing surgery, the intraoperative and postoperative Hb level changes were 7.5 and 8.6 g/L in PKERP and TURP group, as shown in Table 2. No patients required another surgery because of severe postoperative bleeding and all patients did not undergo blood transfusion. During surgery, five patients in PKERP group and four patients in TURP group were found to have narrow urethras and the resectoscope sheaths were only successfully inserted after urethral dilation (*p* > 0.05). This resulted in extended urinary catheter retention time postoperatively. No cases of bladder neck contracture requiring another surgery occurred postoperatively. Thirty-seven patients in PKERP group and 29 patients in TURP group had comorbid bladder stones and underwent transurethral laser lithotripsy for bladder stones (*p* > 0.05).

**Table 2:** Surgical data.

**Items**	**PKERP**	**TURP**	***P***
Decrease in preoperative hemoglobin (Hb) changes (g/L)	7.5 ± 2.3	8.6 ± 2.5	*p* < 0.05
Mean weight of the pathological specimen (g)	37.7 ± 11.2	33.3 ± 9.8	*p* >0.05
Postoperative catheter retention time	5.5 ± 0.7	7.0 ± 0.8	*p* >0.05
Surgical irrigation time(d)	1.7 ± 0.4	2.8 ±0 .6	*p* >0.05
Length of hospitalization	9.1 ± 0.8	9.9 ± 1.1	*p* >0.05
Presence of comorbid bladder stones	37 (30.1%)	29 (20.3%)	*p* >0.05

### Follow-Up Status

Follow-up was done at 3 months, 1 year after the surgical treatment. Routine urine test showed recovery to normal levels in all patients at postoperative month 3. Among these patients, 13 (10.6%) patients still had nocturia ≥ 3 times, whereas 19 (15.44%) patients had nocturia ≤ 1 time. This was considered to be due to long obstruction duration and slow recovery of bladder function after injury. All 123 patients in PKERP could self-control urination and did not experience urinary incontinence (UI). The IPSS, QoL score, and MFR were recorded for each follow-up. As shown in Table 3, no significant differences were detected for the baseline IPSS, QoL score, and MFR between the two groups. The IPSSs of the PKERP group at 3 months and 1 year follow-up were significantly lower than the TURP group. Also, the MFRs of the PKERP group at 1 year follow-up were significantly higher than the TURP group. Further, the QoL scores present no significant differences between the two groups for all the follow-ups. These results showed that PKERP generated better longtime clinical efficacy compared with TURP in elderly patients aged ≥80 years.

**Table 3:** Functional results during follow-up.

**Parameters**	**Preoperative**	**3 months**	**12 months**
IPSS			
PKERP	20.4 ± 2.8	4.8 ± 1.1	4.1 ± 1.3
TURP	23.6 ± 2.3	5.0 ± 1.4	4.9 ± 1.0
P value	*p* > 0.05	*p* < 0.05	*p* < 0.05
QoL			
PKERP	4.6 ± 0.4	2.2 ± 1.5	1.2 ± 0.4
TURP	4.7 ± 0.6	2.3 ± 1.6	2.1 ± 0.7
P value	*p* > 0.05	*p* > 0.05	*p* > 0.05
Qmax (mL/s)			
PKERP	7.4 ± 1.9	18.8 ± 5.8	23.2 ± 2.2
TURP	7.1 ± 1.5	19.6 ± 4.1	19.4 ± 2.9
P value	*p* > 0.05	*p* < 0.05	*p* < 0.05

## Discussions

Transurethral resection of the prostate has a short learning curve and is easy to master. Therefore, it is widely used in primary hospitals. However, accumulated clinical evidence has revealed that there are some disadvantages, such as high intraoperative bleeding, which significantly affects efficacy and outcome of treatment and is not easily controlled. As the prostate volume increases, the operation time and risk of TURS will inevitably increase. This is particularly observed in patients with prostate volume > 80 ml, in this case where the application of TURP is limited. Therefore, local and overseas scholars have doubts on its status as the gold standard (4, 5).

Holmium laser enucleation of the prostate (HoLEP) is currently the most studied and widely used laser prostate enucleation and was first reported by Gilling et al. in 1998. The surgery opens a new field, where the hyperplastic prostate gland is isolated along the capsule surface before a tissue morcellator is used to remove the tissue. Various major clinical guidelines, such as the guidelines of the European Association of Urology and the American Urological Association, have recommended HoLEP as the method to treat prostate hyperplasia. However, the use of this method requires the addition of holmium laser equipment. At the same time, the increased medical expenditure due to high consumption of laser optical fibers remarkably hinders the application of HoLEP, especially in primary hospital.

Transurethral PKERP was first proposed by Professor Chunxiao Liu in 2003 in China. In this surgery, a plasmakinetic loop electrode that was originally used for TURP is used and a resectoscope sheath is used inside the cavity, simulating fingers for complete stripping of a part of the hyperplastic prostate gland along the surgical capsule. Subsequently, a tissue morcellator is used to morcellate the prostate gland before extraction. This technology greatly improves the safety and efficiency of the surgery. At the same time, PERKP does not require additional equipment and is favorable to be conducted in primary hospitals.

The safety of elderly patients during the perioperative phase is an issue that needs a great deal of attention. Among previous perioperative complications of TURP, bleeding and TURP syndrome are particularly serious. The incidence of bleeding and TURP syndrome are associated with the prostate volume, which are 2.0 and 1.2% for prostate volume <30 ml and 9.5% and 3.0% for prostate volume > 60 ml, respectively (6). Elderly patients aged >80 years usually undergo urine retention and ineffective long-term conservative drug treatment for a long time before surgical treatment. From our statistical data, we observed that the mean prostate volume was 67.7 mL and 62.9 mL, and 68.48 and 63.43% of patients had a prostate volume of >80 ml in PKERP and TURP group, respectively. A large prostate volume will increase the operation time and risk of intraoperative bleeding, which significantly increases the risk of surgery. There is less decrease in preoperative hemoglobin change for patients in PKERP group. Moreover, all patients in PKERP group did not receive blood transfusion and did not experience prostatic capsular perforation and TURS syndrome, demonstrating the high safety of PKERP.

Among long-term TURP complications, difficulty in urination is the most common. The risk of re-surgery caused by this complication is 8.3~14.7% on the postoperative 8th year (7). Conversely, the safety and efficacy of HoLEP have been completely validated.

Through nearly 20 years of clinical studies. In a retrospective study with a median follow-up of 62 months, the symptoms, maximum UFR, and residual urine index of 949 patients after treatment of HoLEP within 10 years were significantly improved. In addition, the incidence of long-term postoperative complications was only 0.5–1.6%. Only 0.7% of these patients required re-surgery because of hyperplasia of the remaining prostate gland (8). From our data, we found that even though the equipment used was different, on postoperative day 7, 95% of patients could successfully urinate after their urinary catheters were removed. Only one patient in TURP group was unable to urinate after the urinary catheter was removed. The urinary catheter was retained for another 3 days before removal and this patient could also successfully urinate. Difficulty in urination in this patient was considered to be related to wound edema at the prostatic fossa and poor preoperative detrusor muscle function. UFR examinations were conducted at postoperative months 3 and 12, and MFRs of all patients are mean 18.8 and 23.2 ml/s in PKERP group. Our results were similar to previous data with treatment of HoLEP, demonstrating that surgery outcomes are more associated with surgical method, not equipment. This also suggested that PKERP can effectively treat bladder outlet obstruction (BOO) in elderly patients.

The IPSS and QoL scores reflect the improvement in urination of patients with BPH after treatment. From our retrospective analysis results, it was observed that the postoperative IPSS scores of patients significantly decreased at month 3 in both groups. QoL scores also exhibited similar changes and decreased in two groups. This showed PKERP had similarly immediate effects in alleviating LUTS as TURP. At 3 and 12 months after surgery, it can be seen from the comparison of Table 3 that the improvement of IPSS and MFR was much greater in PKERP group than in TURP group. It can therefore be concluded that PKERP surgery has a stronger durability for improvement of LUTS than TURP.

With regards to influence of surgery on sexual function, we attempted to use the IIEF-5 scale for evaluation and found that 91.00% (212/233) of patients did not have any sexual activity within half a year before consultation. For the reasons, most patients were deeply influenced by traditional Chinese culture and slept on separate beds from their spouses and had low sexual needs. Moreover, severe LUTS also caused distress in the life of patients and affects their sexual needs. The other few patients, who still had sexual activity preoperative, had mild-to-moderate erectile dysfunction. During follow-up at postoperative months 3 and 12, we found that there were no significant changes in the scores after IIEF-5 tests were repeated in PKERP group. To some extent, this shows that PKERP probably has some protective effects on sexual functions due to complete retainment of the prostatic capsule tissues. However, all these patients experienced retrograde ejaculation, showing that PKERP resulted in damage to posterior urethral neuromuscular coordination. Therefore, data from a larger sample size are required for analysis to demonstrate the effects of PKERP on sexual function in elderly patients.

Urodynamic studies test is a reliable method for clinically determining BOO and detrusor muscle status. In our hospital, all elderly patients aged >80 years completed this test preoperatively. From the results, it was observed that 16% of patients had varying degrees of detrusor muscle weakness (maximum detrusor muscle pressure <40 cm H_2_O during urination) preoperatively and 60% of patients had a preoperative bladder capacity of <300 ml. Evaluation of bladder function preoperatively can aid in evaluating surgery outcomes and adopting corresponding management measures in advance. Preoperative communication with patients about the potential outcome of postoperative urination status will significantly relieve their stress and increase their acceptance of surgery. For patients with severe detrusor muscle weakness, it is essential to extend to urinary catheter retention time to facilitate recovering bladder detrusor muscle function. Regarding patients with low bladder capacity, we informed them preoperatively that urinary frequency would persist for some time postoperatively and may require drugs for controlling symptoms. In addition, bladder function training would begin 2 days before the catheter was removed, and the drainage tube would be periodically closed and opened to promote recovery of bladder capacity. These targeted management measures would help alleviation of postoperative symptoms and promote the function recovery.

From the outcome of patients with surgical treatment, there are advantages of PKERP in elderly patients. (1) Reduced bleeding during surgery: The entire enucleation process is conducted under clear vision, and it is an intracavity enucleation along the surgical capsule in the true sense, in which blood vessels are clearly visible and hemostasis can be immediately conducted on evident bleeding from blood vessels or point bleeding. These features are prior to the previous TURP, in which repeated hemostasis is required on the same blood vessel. (2) Intact capsule and perforation are avoided: Under direct view, the capsule structure is extremely clear and is easily identified. With these features, PKERP makes perforation not easily occurred and reduces the incidence of TURS compared with that observed with conventional TURP (9). (3) Complete prostate gland resection: Palaniappan et al. (10) reported that the mean resection rate during TURP is only 45.5%. In our study, the weight of specimens resected by PKERP was significantly higher than that of TURP group. It showed that the prostate enucleation was thorough and efficient. During postoperative follow-up, MFR greatly increased, which suggested that BOO in patients was significantly relieved compared with preoperational. (4) Shortened operation time: As the blood supply to the prostate gland was completely blocked and the surgical field of view was clear, the operation time was greatly shortened. (5) Fewer complications: The most serious complication of prostate enucleation was postoperative stress UI. Prostate volume is a risk factor for UI after prostate enucleation. A study reported that retaining some prostate tissue at the apex of the prostate could reduce the incidence of postoperative UI (11). Therefore, we appropriately retained the apical tissue from 11 to 1 o'clock position for patients with a larger prostate volume to form a “urinary pad.” During surgery, we avoided violently stripping and pushing prostate gland into the bladder. At the same time, there was no need for catheter traction postoperatively, and levator ani muscle training was provided as early as possible. This significantly decreased the incidence of UI after enucleation. During surgery, we completely retained the circular fibers at the bladder neck. This decreased postoperative retrograde ejaculation, erectile dysfunction, and other complications (12).

## Conclusion

In summary, PKERP is a safe and efficacious surgical method for treating BPH in elderly patients.

## Data Availability Statement

The original contributions presented in the study are included in the article/supplementary material, further inquiries can be directed to the corresponding author.

## Author Contributions

JT: performed the experiment. YY and LZ: performed the data analyses and wrote the manuscript. WS: contributed significantly to analysis and manuscript preparation. All authors contributed to the article and approved the submitted version.

## Funding

This work was supported by grant from the Natural Science Foundation of Hunan Province (No. 2018JJ2608).

## Conflict of Interest

The authors declare that the research was conducted in the absence of any commercial or financial relationships that could be construed as a potential conflict of interest.

## Publisher's Note

All claims expressed in this article are solely those of the authors and do not necessarily represent those of their affiliated organizations, or those of the publisher, the editors and the reviewers. Any product that may be evaluated in this article, or claim that may be made by its manufacturer, is not guaranteed or endorsed by the publisher.
